# Reduced clot strength upon admission, evaluated by thrombelastography (TEG), in trauma patients is independently associated with increased 30-day mortality

**DOI:** 10.1186/1757-7241-19-52

**Published:** 2011-09-28

**Authors:** Kristin B Nystrup, Nis A Windeløv, Annemarie B Thomsen, Pär I Johansson

**Affiliations:** 1 Department of Clinical Immunology, Section for Transfusion Medicine, Rigshospitalet, Copenhagen University Hospital, DK-2100 Copenhagen, Denmark; 2 Department of Anesthesia and Trauma Centre, Centre of Head and Orthopedics, Rigshospitalet, Copenhagen University Hospital, DK-2100 Copenhagen, Denmark

**Keywords:** thrombelastography, trauma, coagulopathy, transfusion

## Abstract

**Introduction:**

Exsanguination due to uncontrolled bleeding is the leading cause of potentially preventable deaths among trauma patients. About one third of trauma patients present with coagulopathy on admission, which is associated with increased mortality and will aggravate bleeding in a traumatized patient. Thrombelastographic (TEG) clot strength has previously been shown to predict outcome in critically ill patients. The aim of the present study was to investigate this relation in the trauma setting.

**Methods:**

A retrospective study of trauma patients with an injury severity qualifying them for inclusion in the European Trauma Audit and Research Network (TARN) and a TEG analysis performed upon arrival at the trauma centre.

**Results:**

Eighty-nine patients were included. The mean Injury Severity Score (ISS) was 21 with a 30-day mortality of 17%. Patients with a reduced clot strength (maximal amplitude < 50 mm) evaluated by TEG, presented with a higher ISS 27 (95% CI, 20-34) vs. 19 (95% CI, 17-22), p = 0.006 than the rest of the cohort. Clot strength correlated with the amount of packed red blood cells (p = 0.01), fresh frozen plasma (p = 0.04) and platelet concentrates (p = 0.03) transfused during the first 24 hours of admission. Patients with low clot strength demonstrated increased 30-day mortality (47% vs. 10%, p < 0.001). By logistic regression analysis reduced clot strength was an independent predictor of increased mortality after adjusting for age and ISS.

**Conclusion:**

Low clot strength upon admission is independently associated with increased 30-day mortality in trauma patients and it could be speculated that targeted interventions based on the result of the TEG analysis may improve patient outcome. Prospective randomized trials investigating this potential are highly warranted.

## Introduction

Exsanguination due to uncontrolled bleeding is the leading cause of potentially preventable deaths among trauma patients [1-3]. Upon arrival at the hospital, about one third of all trauma patients present with coagulopathy [4-6], which is associated with increased transfusion requirements, development of multi organ failure and death [5-7]. The presence of coagulopathy will aggravate active hemorrhaging in traumatized patients.

Coagulopathy associated with traumatic injury has historically been described as the result of multiple environmental factors such as acidemia and hypothermia, which have been shown to independently impair blood clotting [4,7,8]. Combined with dilution and consumption of coagulation factors and platelets secondary to fluid administration and bleeding, severe coagulopathy ensues [5,7]. Recently, an early acute traumatic coagulopathy induced by the trauma and hypoperfusion, leading to up-regulation of thrombomodulin and causing the activation of systemic anticoagulant and fibrinolytic pathways, has been described [7].

**Figure 1:**
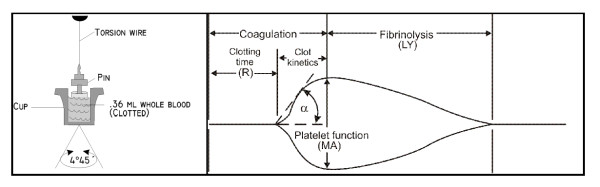
**Thrombelastographic analysis with measured parameters**. Reaction time (R). Alpha angle (α), maximal amplitude (MA), lysis (LY).

Early monitoring of coagulation is essential to identify coagulopathy and this is routinely based on conventional plasma based coagulation tests such as prothrombin time (PT), activated partial thromboplastin time (APTT), international normalized ratio (INR), fibrinogen and platelet number [3]. These plasma-based tests only reflect the initiation of the hemostatic process [9] and they cannot be used for evaluating the amplification of propagation parts or increased fibrinolysis [7]. Thrombelastography (TEG)/thrombelastometry (ROTEM) analyses the viscoelastic properties of whole blood, thereby reflecting the entire hemostatic process. TEG therefore allows evaluation of coagulation in whole blood and comprises a more qualitative analysis of the individual cellular components and their interactions [10-12]. Viscoelastic hemostatic assays such as TEG are currently recommended by several international guidelines and teaching books concerning massive transfusion in trauma [3,13,14]. Furthermore, TEG is the only test available for rapid identification of hyperfibrinolysis, which is associated with increased mortality in trauma patients [15-17]. A schematic TEG trace is shown in Figure 1. In TEG, the hemostatic process is described by several measurements: R (reaction) time is the period from the beginning of the test until clot formation begins. α-angle reflects the increase in clot strength over time, and the maximal amplitude (MA) is a direct measure of the highest point on the TEG curve, representing maximal clot strength. Ly30 is a measurement of the fibrinolytic activity during the first 30 minutes after MA. MA is influenced primarily by platelet concentration and function [10,11]. Low MA has been reported to be the best TEG indicator of increased transfusion requirements and mortality [18,19]. Transfusion therapy guided by TEG has been shown to reduce peri- and postoperative bleeding and transfusion requirements, thereby improving survival rates for massively bleeding patients [20].

In trauma, TEG has been described as a better predictor of transfusion requirements than PT, INR or APTT [18] and recently, initial experiences with TEG-guided management of life-threatening post-injury coagulopathy was reported by Kashuk and colleagues with a favourable outcome when comparing with historic controls [21]. These results align with Schöchl et al. who found a beneficial effect of goal-directed resuscitation in actively bleeding trauma patients when juxtaposed to the patient outcome predicted by trauma and injury severity score (TRISS) [22]. With the consistent findings that low clot strength is associated with poor outcome in trauma patients [15,18], this end-point together with transfusion requirements have been chosen in the present study concerning patients suffering major trauma.

## Methods

A retrospective study including trauma patients from 2006 and 2007, who were admitted at the Trauma Centre at Rigshospitalet, Copenhagen. The inclusion criteria were: The patients should be included in the European Trauma Audit and Research Network (TARN) [23,24], and have a TEG analysis performed along with the initial blood tests sampled upon arrival at the hospital, which occurs before any blood products are administered. TARN is a joint European database containing uniform reports on all included trauma patients. Only including patients with severe traumatic injuries, this database provides access to comparable patient data in the specified time period. Data was collected using several databases. Gender and age were registered using the Danish civil registration numbers. A review of patient charts and TARN-records revealed mechanism and type of traumatic injury and Injury Severity Score (ISS) [23,25]. TEG was performed in the blood bank on citrated blood samples within 5-7 min of blood sampling in accordance with our previously published experience [26]. The TEG analysis was displayed in real-time in the trauma center allowing for immediate interpretation and intervention as described elsewhere [27]. TEG (TEG 5000, Haemoscope, Niles, IL) parameters of R-time, α-angle and maximal amplitude (MA) and laboratory parameters such as hemoglobin and platelet count (Sysmex 2100, Sysmex Corp., Kobe, Japan), APTT and INR (ACL TOP, Beckman Coulter Inc., Brea, CA), lactate and blood glucose (Modular P-modul, Hitachi, Tokyo, Japan) including the number and time for delivery of packed red blood cells (RBC), fresh frozen plasma (FFP) and platelet concentrates (PC) were collected using the Regional Blood Bank Database. Data on hospital length of stay and 30-day mortality was collected using the hospital registration system.

Patients were categorized as having reduced clot strength if MA was lower than the cut-off value supplied from the manufacturer at time of analysis, MA < 50 mm.

### Statistics

Distributions of data were inspected using probability plots, and non-normal distributed variables were log-transformed. Data on patients stratified according to MA < 50 mm were compared by student-T test. Associations of mortality and MA < 50 mm, APTT, blood glucose, hemoglobin, blood lactate, INR or platelet counts were analyzed by multivariable regression including co-variables of age and ISS, using 30-day mortality (yes/no) as dependent variable. Values are presented as means with 95% Confidence Intervals and results of multivariable regression analyses are presented as Odds Ratio (OR) with X^2^. Area under the curve (AUC) using Receiver Operating Characteristic (ROC) was calculated to compare relative prognostic efficiency for 30-day mortality on each assay. P-values < 0.05 were considered significant. Statistical calculations were performed using SPSS 17 (IBM Corp., Somers, NY).

## Results

Demographics of the 89 included patients are presented in Table 1. The vast majority presented with blunt injuries, and 65 out of the 89 patients suffered injuries relating to traffic accidents. The mean ISS of the cohort was 21 and the 30-day mortality was 17%.

**Table 1:** Patient demographics (*n *= 89)

Age (years)	39 (35-43)
Males	59 (66%)

Blunt trauma	76 (85%)

Cause of trauma	

Traffic accident	65

Fall from heights	10

Assault	10

Suicide attempt	4

Type of trauma	

Thoracic	15

Abdominal	7

Extremities	4

Cerebral	18

Multi trauma	37

Other	8

Injury Severity Score	21 (19-23)

Transfusions in units	

RBC	3.4 (2.6-4.4)

FFP	2.2 (1.7-2.8)

PC	1.6 (1.4-1.9)

Length of stay (days)	10.3 (8.1-13.1)

Thirty-day mortality	15 (17%)

Demographics of the 89 patients included in the study. Frequencies are stated as count with percentage in parenthesis. Age, Injury Severity Score, blood product transfusions and length of stay are stated as means with 95% Confidence Intervals. Packed red blood cells (RBC), fresh frozen plasma (FFP) and platelet concentrates (PC).

Patients with reduced clot strength presented with higher ISS 27 (95% CI, 20-34) vs. 19 (95% CI, 17-22), p = 0.006 than the rest of the cohort.

With regards to standard laboratory parameters, hemoglobin and platelet counts were lower in patients with low clot strength compared to the rest of the cohort [9.7 (95% CI 8.5-10.7) vs. 11.9 (95% CI, 11.5-12.4) g/dL, p < 0.001 and 140 (95% CI, 112-168) vs. 214 (95% CI, 196-231) × 10^9^/L, p < 0.001, respectively]. APTT and INR were higher in patients with reduced clot strength when compared to the rest of the cohort [49 (95% CI, 37-66) vs. 29 (95% CI, 27-30) seconds, p < 0.001 and 1.4 (95% CI, 1.3-1.5) vs. 1.2 (95% CI, 1.1-1.2) arbitrary units, p < 0.001, respectively]. Glucose was also higher in patients with low clot strength [10.3 (95% CI, 8.7-12.1) vs. 7.7 (95% CI, 7.2-8.3) mmol/L, p = 0.001].

**Figure 2:**
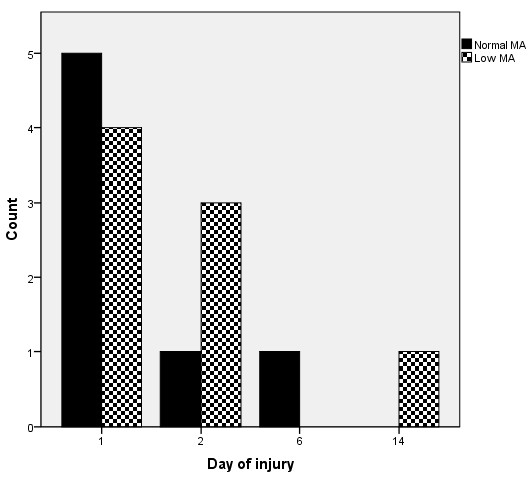
**Mortality in patients with low vs. non-low clot strength**.

During the first 24 hours of admission, patients with reduced clot strength received approximately twice as many blood products compared with the rest of the cohort (Table 2), and there was a significant correlation between clot strength and the amount of transfused RBC (p = 0.01), FFP (p = 0.04), and PC (p = 0.03). Patients presenting with low clot strength demonstrated increased 30-day mortality (47% vs. 10%, p < 0.001). In patients with low clot strength, 7 out of 8 patients expired within the first 48 hours from hospital admission (Figure 2).

**Table 2:** Patients stratified according to clot strength

	Low clot strength *n = 17*	Normal or high clot strength *n = 72*	p-value
Age (years)	43 (32-54)	38 (34-43)	0.38

Males	10 (59%)	49 (68%)	0.47

Blunt trauma	16 (94%)	60 (83%)	0.45

Type of trauma			0.66

Thoracic	1	14	

Abdominal	1	6	

Extremities	0	4	

Cerebral	5	13	

Multi trauma	8	29	

Other	2	6	

Injury Severity Score	27 (20-34)	19 (17-22)	0.006

Laboratory analyses			

Hemoglobin (g/dL)	9.7 (8.5-10.7)	11.9 (11.5-12.4)	<0.001

Platelet count (10^9^/L)	140 (112-168)	214 (196-231)	<0.001

APTT (seconds)	49 (37-66)	29 (27-30)	<0.001

INR (arbitrary units)	1.4 (1.3-1.5)	1.2 (1.1-1.2)	<0.001

Glucose (mmol/L)	10.3 (8.7-12.1)	7.7 (7.2-8.3)	0.001

Lactate (mmol/L)	3.3 (2.4-4.6)	2.5 (2.1-2.9)	0.11

TEG			

R (minutes)	6.3 (5.2-7.4)	5.2 (4.9-5.5)	0.01

Angle (degrees)	47 (40-53)	65 (63-67)	0.001

MA (mm)	39 (34-44)	60 (59-61)	N/A

Ly30 (%)	0.8 (0.0-1.7)	0.7 (0.5-0.9)	0.58

Transfusions in units			

RBC	6.4 (3.7-11.2)	2.9 (2.2-3.8)	0.02

FFP	3.6 (1.9-6.6)	2.0 (1.5-2.6)	0.06

PC	2.3 (1.5-3.6)	1.5 (1.2-1.7)	0.04

Length of stay (days)	4.9 (2.7-8.7)	12.3 (9.6-15.7)	0.002

Data of patients with low clot strength (maximal amplitude < 50 mm) compared to patients with normal or high clot strength (maximal amplitude ≥ 50 mm). Frequencies are stated as count with percentage in parenthesis. Age, laboratory and thrombelastographic (TEG) analyses, blood product transfusions, Injury Severity Score and length of stay are stated as means with 95% Confidence Intervals. Activated partial thromboplastin time (APTT), international normalized ratio (INR), packed red blood cells (RBC), fresh frozen plasma (FFP), platelet concentrates (PC).

By logistic regression analysis with 30-day mortality as endpoint, reduced clot strength was an independent predictor of increased mortality after adjusting for age and ISS (Table 3).

**Table 3:** Prediction of mortality by low clot strength, age and Injury Severity Score

	OR	95% CI	p-value	X^2^
Injury Severity Score (per point)	1.09	(1.01-1.16)	0.02	6.6

Age (per year)	1.03	(1.00-1.06)	0.09	3.0

Low clot strength (maximal clot strength < 50 mm)	5.00	(1.22-20.45)	0.03	4.8

Multivariable analysis of age, Injury Severity Score and low clot strength (maximal amplitude < 50 mm) with 30-day mortality as dependent variable.

Likewise, APTT (OR = 1.1 (95% CI, 1.0-1.2), chi-square = 18.8, p = 0.008), glucose (OR = 1.4 (95% CI, 1.1-1.8), chi-square = 15.6, p = 0.002) and lactate (OR = 1.4 (95% CI, 1.1-1.9), chi-square = 8.5, p = 0.007) were also associated with 30-day mortality after adjusting for age and ISS.

**Figure 3:**
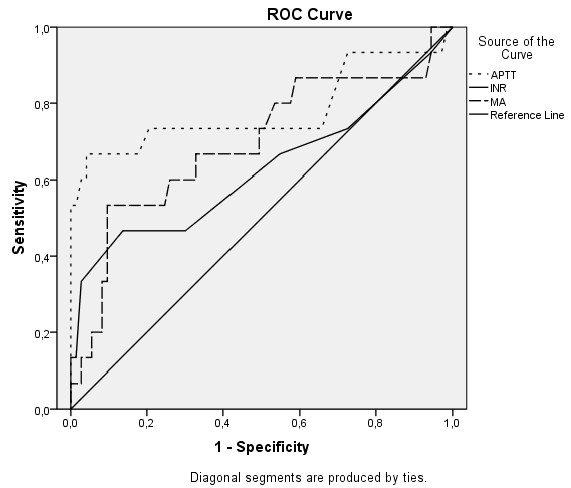
**ROC curves for APTT, INR and MA in relation to mortality**. Activated partial thromboplastin time (APTT), international normalized ratio (INR), maximal amplitude (MA).

Relative prognostic efficiency for 30-day mortality using ROC AUC was for APTT 0.78 (95% CI, 0.61-0.95) p < 0.001, for INR 0.63 (95% CI, 0.44-0.81) p = 0.13 and for MA 0.70 (95% CI, 0.53-0.86) p = 0.02 (Figure 3).

Only one patient presented with pathologically increased fibrinolysis, 54% (normal < 8%). This patient also demonstrated reduced clot strength, had the highest ISS in the cohort (66) and expired on the day of admission.

## Discussion

The main finding of the present study was that low clot strength evaluated by TEG was independently associated with increased mortality at 30-days post trauma, also after adjusting for ISS and age. To our knowledge, this is the first study reporting of an independent association between reduced clot strength upon admission and 30-day mortality in trauma patients. Recently, Kashuk et al. found a similar association between low clot strength and 24-hour survival in trauma patients [21]. Our results correspond to the findings of Carroll et al., who in 161 trauma patients found that non-survivors presented with significantly lower clot strength compared to survivors [15]. Abnormally reduced clot strength has also previously been reported to be associated with increased 30-day mortality in patients admitted to the intensive care unit, reflecting the clinical significance of whole blood viscoelastic hemostatic assays such as TEG in critically ill patients [19]. The association between reduced clot strength upon hospital admission, increased transfusion requirements and high 30-day mortality may reflect that patients with reduced clot strength more frequently develop life-threatening bleedings and thereby experience more episodes of hypoperfusion. This is indicated by the increased lactate in these patients when compared to those with normal clot strength. Consequently, normalizing hemostasis in these patients may improve outcome. In alignment with this, we have previously demonstrated that early aggressive administration of plasma and platelets in addition to RBC can reverse the acute coagulopathy of trauma found by TEG [26]. This transfusion strategy reduces mortality in massively bleeding patients [20], indicating that goal-directed therapy based on TEG may improve outcome. This corresponds to the findings of Kashuk et al., who reported that using TEG for management of life-threatening postinjury coagulopathy was associated with a favourable outcome when compared to historic controls [21].

An independent association between APTT and 30-day mortality aligns well with the findings of Brohi et al. and Macleod et al. who reported that a substantial proportion of trauma patients upon admission presented with coagulopathy unrelated to resuscitation fluids and hypothermia, and that this was associated with a 3-4 fold increased mortality [4,6]. Interestingly, plasma based coagulation assays have consistently been shown not to correlate to relevant clinical bleeding conditions [28] and consequently, in trauma patients, mild prolongation of APTT and/or INR must reflect something else of relevance for outcome in these severely injured patients. In the present study, APTT demonstrated the best ROC characteristics with regard to mortality, and we speculate that this reflects the degree of endothelial breakdown [29]. Supporting this, we recently found that severely injured trauma patients presented with increased glycocalyx and endothelial breakdown, evaluated by syndecan-1 and soluble thrombomodulin (sTM) in plasma, respectively. Constituents of the glycocalyx such as heparansulphate and syndecan-1 together with endothelial sTM all have potent anticoagulant properties, and this may be reflected by increased APTT [30].

Brohi and coworkers coined the term ACoTS to describe this acute coagulopathy of trauma [31]. It has been postulated that the most important factors leading to this condition are tissue injury and shock, and that the coagulopathy identified by increased APTT and PT is a result of activation of the protein C system together with increased fibrinolysis [32]. The importance of tissue hypoperfusion for outcome of trauma patients, as suggested by Brohi et al. [7], corresponds well with our finding of lactate being independently associated with 30-day mortality also after adjusting for ISS and age. The relevance of blood lactate for mortality in trauma patients was recently reported by Vandromme et al. [33]. When comparing patients with reduced clot strength to the rest of the cohort, the hypocoagulable patients were more seriously injured as reflected by a higher ISS. Carroll et al. and Kaufmann et al. also reported that trauma patients with evidence of TEG hypocoagulability had higher ISS than those presenting with normal or hypercoagulable TEG profiles [15,34]. The hypocoagulable TEG may reflect increased consumption of coagulation factors and platelets secondary to the trauma, thus displaying disseminated intravascular coagulation (DIC) with a hemorrhagic phenotype [35] or, as discussed previously, ACoTS [7], or perhaps a combination. The standard coagulation analyses such as APTT and INR were prolonged in TEG hypocoagulable patients, and this may be ascribed to both DIC with a hemorrhagic phenotype and ACoTS, whereas the reduced platelet count in hypocoagulable patients might indicate a consumptive state.

Hyperfibrinolysis has been reported to be an integral part of the coagulopathy of trauma [36] and Schöchl et al. has reported that this condition is observed in the most seriously injured patients, and is associated with elevated mortality [16]. This corresponds to the findings of the present study, where only the patient with the highest ISS of the entire cohort presented with increased fibrinolysis.

In the present study, patients with hypocoagulable TEG received twice as many blood product transfusions during the first 24 hours of admission than the rest of the cohort. A significant correlation between clot strength and the amount of transfused RBC, FFP and PC was found. Plotkin et al. reported that in combat trauma patients, thrombelastography was a more accurate indicator of blood product requirements than PT, APTT and INR [18]. Furthermore, Kaufmann et al. found that only ISS and TEG were predictive of transfusions, whereas PT and APTT were not [34]. The superiority of TEG in identifying clinically relevant coagulopathies and blood product requirements can be explained by the introduction of the cell-based model of hemostasis. This emphasizes the role of platelets for intact thrombin generation and highlights the importance of the dynamics in thrombin generation, which affect the quality and stability of the thrombus formed [37]. Consequently, hemostatic assays performed on plasma such as APTT and PT are of limited value [38] and do not correlate with clinically relevant coagulopathies or bleeding conditions [28]. To date, more than 25 studies including more than 4,500 patients have evaluated TEG versus conventional coagulation assays on bleeding and transfusion requirements in surgical patients undergoing cardiac, liver, vascular or trauma surgery and in patients requiring massive transfusion. These studies all report that whole blood TEG is superior in predicting the need for blood transfusion, and that treatment based on the results of the TEG analysis reduces transfusion requirements and the need for re-do surgery in contrast to treatments relying on plasma based coagulation assays [11]. Early identification and institution of goal-directed treatment of post-traumatic coagulopathy could, potentially, improve outcome in trauma patients as indicated in the studies performed by Kashuk et al. and Schöchl et al. [21,22].

Our results are subject to limitations inherent to observational studies and thereby do not allow independent estimation of the cause-and-effect relationship between the TEG results and outcome. The results of this study indicate an association rather than a correlation between low clot strength and mortality post-trauma. Furthermore, this is a retrospective study, which was conducted in a limited number of patients at a single centre and, although internal validity is high, external validity may be limited. Another limitation of the present study is, that the reported changes of TEG parameters had to be interpreted on the basis of external reference values. The clinical inhibitory effect of antithrombotic medications such as clopidogrel and aspirin on platelet aggregation cannot be assessed using hemostatic assays, because the assay activators cancel this inhibition. Nor will conditions affecting the endothelium such as von Willebrand disease be detected by hemostatic assays [11].

## Conclusions

Low clot strength in trauma patients, evaluated by TEG upon admission to the trauma centre is independently associated with increased 30-day mortality, even after adjusting for age and ISS. We believe that targeted interventions with plasma and platelets in addition to RBC together with antifibrinolytic therapy, based on the results of the TEG analysis, may improve outcome in trauma patients. Prospective randomized trials investigating this potential are highly warranted.

## Competing interests

The authors declare that they have no competing interests.

## Authors' contributions

KN performed all data collection. KN, PJ conducted MEDLINE searches for relevant publications related to thrombelastography and coagulopathy in trauma, and by review of searched articles jointly decided which to include. KN, PJ wrote the first draft of the manuscript. NW made the statistical analyses and designed the tables. All authors read and approved the final manuscript.

## References

[B1] Sauaia A Moore FA Moore EE Moser KS Brennan R Read RA Pons PT Epidemiology of trauma deaths: a reassessment J Trauma 1995 38 2 185 193 10.1097/00005373-199502000-00006 7869433

[B2] Eastridge BJ Malone D Holcomb JB Early predictors of transfusion and mortality after injury: A review of the data-based literature J Trauma 2006 60 Suppl 6 S20 25 16763476 10.1097/01.ta.0000199544.63879.5d

[B3] Rossaint R Bouillon B Cerny V Coats TJ Duranteau J Fernández-Mondéjar E Hunt BJ Komadina R Nardi G Neugebauer E Ozier Y Riddez L Schultz A Stahel PF Vincent JL Spahn DR Management of bleeding following major trauma: an updated European guideline Crit Care 2010 14 2 R52 10.1186/cc8943 20370902 PMC2887168

[B4] Brohi K Singh J Heron M Coats T Acute traumatic coagulopathy J Trauma 2003 54 6 1127 1130 10.1097/01.TA.0000069184.82147.06 12813333

[B5] Maegele M Lefering R Yucel N Tjardes T Rixen D Paffrath T Simanski C Neugebauer E Bouillon B Early coagulopathy in multiple injury: an analysis from the German Trauma Registry on 8724 patients Injury 2007 38 3 298 304 10.1016/j.injury.2006.10.003 17214989

[B6] Macleod JBA Lynn M McKenney MG Cohn SM Murtha M Early coagulopathy predicts mortality in trauma J Trauma 2003 55 l 39 44 12855879 10.1097/01.TA.0000075338.21177.EF

[B7] Brohi K Cohen MJ Davenport RA Acute coagulopathy of trauma: mechanism, identification and effect Curr Opin Crit Care 2007 13 6 680 685 10.1097/MCC.0b013e3282f1e78f 17975390

[B8] Watts DD Trask A Soeken K Perdue P Dols S Kaufmann C Hypothermic coagulopathy in trauma: effect of varying levels of hypothermia on enzyme speed, platelet function, and fibrinolytic activity J Trauma 1998 44 5 846 854 10.1097/00005373-199805000-00017 9603087

[B9] Mann KG Butenas S Brummel K The dynamics of thrombin formation Arterioscler Thromb Vasc Biol 2003 23 1 17 25 10.1161/01.ATV.0000046238.23903.FC 12524220

[B10] Reikvam H Steien E Hauge B Liseth K Hagen KG Størkson R Hervig T Thrombelastography Transfus Apher Sci 2009 40 2 119 123 10.1016/j.transci.2009.01.019 19249246

[B11] Johansson PI Stissing T Bochsen L Ostrowski SR Thrombelastography and tromboelastometry in assessing coagulopathy in trauma Scand J Trauma Resusc Emerg Med 2009 17 45 52 10.1186/1757-7241-17-45 19775458 PMC2758824

[B12] Rugeri L Levrat A David JS Delecroix E Floccard B Gros A Allaouchiche B Negrier C Diagnosis of early coagulation abnormalities in trauma patients by rotation thrombelastography J Thromb Haemost 2007 5 2 289 295 10.1111/j.1538-7836.2007.02319.x 17109736

[B13] American Society of Anesthesiologists Task Force on Perioperative Blood Transfusion and Adjuvant Therapies Practice guidelines for perioperative blood transfusion and adjuvant therapies: an updated report by the American Society of Anesthesiologists Task Force on Perioperative Blood Transfusion and Adjuvant Therapies Anesthesiology 2006 105 1 198 208 10.1097/00000542-200607000-00030 16810012

[B14] Hess JR Johansson PI Holcomb JB Mintz PD Trauma and massive transfusion Transfusion Therapy: Clinical Principles and Practice 2010 3 Bethesda, MD: AABB Press 305 322

[B15] Carroll RC Craft RM Langdon RJ Clanton CR Snider CC Wellons DD Dakin PA Lawson CM Enderson BL Kurek SJ Early evaluation of acute traumatic coagulopathy by thrombelastography Transl Res 2009 154 1 34 39 10.1016/j.trsl.2009.04.001 19524872

[B16] Schöchl H Frietsch T Pavelka M Jámbor C Hyperfibrinolysis after major trauma: Differential diagnosis of lysis patterns and prognostic value of thrombelastometry J Trauma 2009 67 1 125 131 10.1097/TA.0b013e31818b2483 19590321

[B17] Kashuk JL Moore EE The emerging role of rapid thromboelastography in trauma care J Trauma 2009 67 2 417 418 19667905 10.1097/TA.0b013e3181ac9cdc

[B18] Plotkin AJ Wade CE Jenkins DH Smith KA Noe JC Park MS Perkins JG Holcomb JB A reduction in clot formation rate and strength assessed by thrombelastography is indicative of transfusion requirements in patients with penetrating injuries J Trauma 2008 64 Suppl 2 S64 68 18376174 10.1097/TA.0b013e318160772d

[B19] Johansson PI Stensballe J Windeløv N Perner A Espersen K Hypocoagulability, as evaluated by thrombelastography, at admission to the ICU is associated with increased 30-day mortality Blood Coagul Fibrinolysis 2010 21 2 168 174 10.1097/MBC.0b013e3283367882 20051844

[B20] Johansson PI Stensballe J Effect of haemostatic control resuscitation on mortality in massively bleeding patients: a before and after study Vox Sang 2009 96 2 111 118 10.1111/j.1423-0410.2008.01130.x 19152603 PMC2667686

[B21] Kashuk JL Moore EE Wohlauer M Johnson JL Pezold M Lawrence J Biffl WL Burlew CC Barnett C Sawyer M Sauaia A Initial experiences with point-of-care rapid thrombelastography for management of life-threatening postinjury coagulopathy Transfusion 2011 in press 10.1111/j.1537-2995.2011.03264.x 21790635

[B22] Schöchl H Nienaber U Hofer G Voelckel W Jambor C Scharbert G Kozek-Langenecker S Solomon C Goal-directed coagulation management of major trauma patients using thromboelastometry (ROTEM)-guided administration of fibrinogen concentrate and prothrombin complex concentrate Crit Care 2010 14 2 R55 10.1186/cc8948 20374650 PMC2887173

[B23] Edwards A Di Bartolomeo S Chieregato A Coats T Della Corte F Giannoudis P Gomes E Groenborg H Lefering R Leppaniemi A Lossius HM Ortenwal P Roise O Rusnak M Sturms L Smith M Thomsen AB Willett K Woodford M Yates D Lecky F A comparison of European Trauma Registries. The first report from the EuroTARN Group Resuscitation 2007 75 2 286 297 17714850 10.1016/j.resuscitation.2007.06.023

[B24] The Trauma Audit and Research Network - Procedures Manual https://www.tarn.ac.uk/content/downloads/53/Procedures%20manual.pdf search date 01.05.2009

[B25] Baker SP O'Neill B Haddon W Jr Long WB The injury severity score: a method for describing patients with multiple injuries and evaluating emergency care J Trauma 1974 14 3 187 196 10.1097/00005373-197403000-00001 4814394

[B26] Johansson PI Bochsen L Stensballe J Secher NH Transfusion packages for massively bleeding patients: the effect on clot formation and stability as evaluated by Thrombelastograph (TEG) Transfus Apher Sci 2008 39 l 3 8 18583193 10.1016/j.transci.2008.05.012

[B27] Johansson PI Goal-directed hemostatic resuscitation for massively bleeding patients: the Copenhagen concept Transfus Apher Sci 2010 43 3 401 405 10.1016/j.transci.2010.09.002 20951650

[B28] Segal JB Dzik WH Paucity of studies to support that abnormal coagulation test results predict bleeding in the setting of invasive procedures: an evidence-based review Transfusion 2005 45 9 1413 1425 10.1111/j.1537-2995.2005.00546.x 16131373

[B29] Johansson PI Ostrowski SR Acute coagulopathy of trauma: Balancing progressive catecholamine induced endothelial activation and damage by fluid phase anticoagulation Med Hypotheses 2010 75 6 564 567 10.1016/j.mehy.2010.07.031 20708846

[B30] Johansson PI Stensballe J Rasmussen LS Ostrowski SR A high admission syndecan-1 level, a marker of endothelial glycocalyx degradation, is associated with inflammation, protein C depletion, fibrinolysis, and increased mortality in trauma patients Ann Surg 2011 254 2 194 200 10.1097/SLA.0b013e318226113d 21772125

[B31] Hess JR Lawson JH The coagulopathy of trauma versus disseminated intravascular coagulation J Trauma 2006 60 Suppl 6 S12 19 16763475 10.1097/01.ta.0000199545.06536.22

[B32] Frith D Goslings JC Gaarder C Maegele M Cohen MJ Allard S Johansson PI Stanworth S Thiemermann C Brohi K Definition and drivers of acute traumatic coagulopathy: clinical and experimental investigations J Thromb Haemost 2010 8 9 1919 1925 10.1111/j.1538-7836.2010.03945.x 20553376

[B33] Vandromme MJ Griffin RL Weinberg JA Rue LW Kerby JD Lactate is a better predictor than systolic blood pressure for determining blood requirement and mortality: could prehospital measures improve trauma triage? J Am Coll Surg 2010 210 5 861 869 10.1016/j.jamcollsurg.2010.01.012 20421067

[B34] Kaufmann CR Dwyer KM Crews JD Dols SJ Trask AL Usefulness of thrombelastography in assessment of trauma patient coagulation J Trauma 1997 42 4 716 720 discussion 720-722 10.1097/00005373-199704000-00023 9137263

[B35] Sawamura A Hayakawa M Gando S Kubota N Sugano M Wada T Katabami K Disseminated intravascular coagulation with a fibrinolytic phenotype at an early phase of trauma predicts mortality Thromb Res 2009 124 5 608 613 10.1016/j.thromres.2009.06.034 19660788

[B36] Kashuk JL Moore EE Sawyer M Wohlauer M Pezold M Barnett C Biffl WL Burlew CC Johnson JL Sauaia A Primary fibrinolysis is integral in the pathogenesis of the acute coagulopathy of trauma Ann Surg 2010 252 3 434 442 discussion 443-444 20739843 10.1097/SLA.0b013e3181f09191

[B37] Roberts HR Hoffman M Monroe DM A cell-based model of thrombin generation Semin Thromb Hemost 2006 32 Suppl l 32 38 16673264 10.1055/s-2006-939552

[B38] Fries D Innerhofer P Schobersberger W Time for changing coagulation management in trauma-related massive bleeding Curr Opin Anaesthesiol 2009 22 2 267 274 10.1097/ACO.0b013e32832678d9 19390253

